# Allylphenoxypiperidinium halides as corrosion inhibitors of carbon steel and biocides

**DOI:** 10.3906/kim-2001-23

**Published:** 2020-06-01

**Authors:** Gunay MEHDIYEVA MUZAKIR, Musa BAIRAMOV RZA, Shahnaz HOSSEINZADEH BAHADOR, Gulnara HASANOVA MUSA

**Affiliations:** 1 Department of Chemistry, Faculty of Chemistry, Baku State University, Baku Azerbaijan

**Keywords:** Piperidinium halides, phenolic compounds, corrosion inhibitors, adsorption, biocides

## Abstract

A series of 1-(4-(2-allylphenoxy)butyl)piperidin-1-ium halides (4a-d) was synthesized and characterized via spectroscopic methods (FTIR, 1 H NMR). The corrosion inhibition of the synthesized halides on carbon steel in water-salt-hydrocarbon environment, saturated with H2 S, was investigated. For this purpose, a series of techniques such as gravimetric measurement, potentiodynamic polarisation, and scanning electron microscope (SEM) were used and some thermodynamic parameters of corrosion process (Δ *G_ads._* , *ΔH^0^_ads._* , Δ *S^0^_ads._* ) were evaluated. The steel surface was checked by SEM, and the steel surface showed good surface coverage. The results showed that the synthesized compounds at the concentrations 125, 150 mg ×L^-1^ have corrosion inhibition activity of 78%–95% by gravimetric measurements and 81%–92% by potentiodynamic measurements at 100, 150 mg ×L^-1^. The biological activity was examined against sulphate-reducing bacteria (SRB). It was revealed that at the concentration of compounds 4c and 4d, 100 mg ×L^-1^, the antibacterial activity was 100%.

## 1. Introduction

The protection of different equipment from corrosion is one of the most important ecological and economic problems of petrochemistry, especially in environments containing hydrogen sulphide, CO2 , salt, and other substances [1–5]. Moreover, the corrosion of metal constructions in oil, gas and gas condensate fields due to sulphate-reducing bacteria (SRB), which is mainly carried out with the formation of hydrogen sulphide and other sulphides [6–10], is a serious problem.

It is worth noting that a large number of organic compounds with anticorrosive, bactericidal, and other properties have been discovered [11–26]. While using such compounds as corrosion inhibitors, the following can be observed: the formation of chemisorbed layers on the metal surface and a sharp decrease in the rate of corrosion. Although the various corrosion inhibitors comprising structure functional fragments and heteroatoms (O, N, S, P) are known [27], organic compounds with anticorrosive and bactericidal (SRB) properties are very limited [8,28]. Therefore, it is crucial to work towards discovering new corrosion inhibitors with these characteristics.

It is worth noting that alkenylphenol and their derivatives are functionally substituted organic compounds [29–42] that can be applied as effective corrosion inhibitors and biocides [28, 43–45]. Furthermore, such compounds can be used as light stabilisers for polymers [46], additives in mineral oils and fuels [47-49], to obtain photo-resisting materials [50,51]. Cationic forms of alkenylphenol derivatives are especially important as corrosion inhibitors and biocides, as they are highly soluble in water [28,44].

This paper aims to discover new corrosion inhibitors through the synthesis of new allylphenol derivatives containing nitrogen, halides, and unsaturated fragments in their structures. This will be accomplished by studying these new allylphenol derivatives as biocides and inhibitors of the hydrogen-sulphide corrosion of St.3 steel, employing gravimetric (25 °C, 35 °C, and 45 °C) and electrochemical (25 °C) methods in systems comprising a 3% aqueous solution of NaCl and hydrocarbons (kerosene) (water: kerosene = 9:1 vol.) saturated with hydrogen sulphide. The thermodynamic parameters (ΔG, ΔH, and ΔS) of corrosion in identical conditions in the presence of synthesized inhibitors were determined.

## 2. Materials and methods

### 2.1. Synthesis

#### 2.1.1. Synthesis of 1-allyl-2-(4-bromobutoxy)benzene (2)

1-allyl-2-(4-bromobutoxy)benzene (2) was obtained [52] by interaction of 13.4 g (0.1 mol) 2-allylphenol (Aldrich) (1) with 43.2 g (0.2 mol) 1,4-dibromobutane (Aldrich) in 2-propanol (as a solvent) (Karmalab) in the presence of 5.6 g (0.1 mol) KOH (Karmalab). The reaction carried out at 80 °C for 1.5 h (Scheme). bp = 130 °C /5 mm, n^20^_D_ = 1.530, d^20^_4_ = 1.242 g× cm^-3^.

**Scheme:**
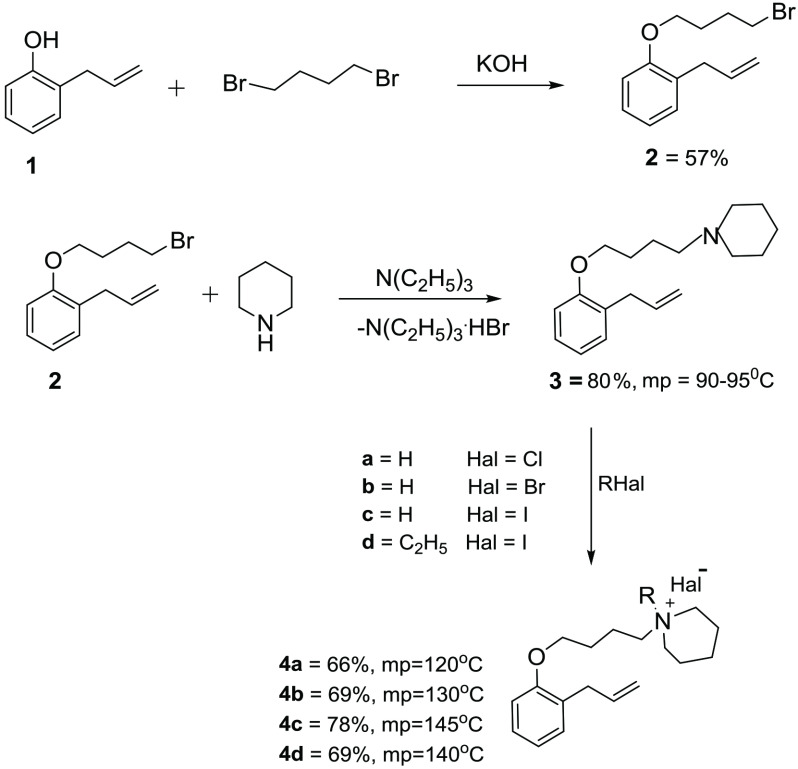
The structures of the synthesized compounds 4a-d.

#### 2.1.2. Synthesis of 1-(4-(2-allylphenoxy)butyl) piperidine (3)

1-(4-(2-Allylphenoxy)butyl) piperidine (3) was obtained by a reaction of equimolar of 1-allyl-2-(4-bromobutoxy) benzene (2) amounts and piperidine (Karmalab) in the presence of triethylamine (Karmalab) (Scheme). The reaction was carried out at 25 °C for 24 h. The obtained mixture was filtered and dried with Na_2_SO_4_ (ECOS). It was then distilled under high vacuum due to separate unreacted starting reagents.

#### 2.1.3. Synthesis of ammonium salts 4a-d

Piperidinium halides (4a-d) were prepared with a reaction of equimolar amounts 1-(4-(2-allylphenoxy)butyl) piperidine (3) with hydrogen halides (HCl, HBr, and HI) and ethyl iodide at 30 °C–40 °C for a reaction period 0.5 h. Hydrogen halides were obtained by the reaction of NaCl, KBr, KI (Karmalab) with sulfuric acid (Karmalab). The obtained 1-(4-(2-allylphenoxy)butyl)-piperidin-1-ium chloride (4a), 1-(4-(2-allylphenoxy)butyl)-piperidin-1-ium bromide (4b), 1-(4-(2-allylphenoxy)butyl)-piperidin-1-ium iodide (4c) and 1-(4-(2-allylphenoxy)butyl)-1-ethylpiperidin-1-ium iodide (4d) (Scheme) were recrystallised several times from water and then dried under vacuum.

The structure of the prepared compounds 4a, b, d and compound 2,3 were confirmed by the FTIR and ^1^H NMR spectroscopy method. For the FTIR analysis, “Varian 3600 FTIR” were used for NMR-spectroscopy–BRUKER FT NMR spectrometer AVANCE 300 (300 MHz) with a BVT 3200 variable temperature unit in 5-mm sample tubes, using Bruker Standard software (solvent–D_2_O, CDCl_3_).

### 2.2. Gravimetric corrosion tests

The gravimetric measurements were carried out at 25 °C, 35 °C, and 45 °C for 5 h in a mixture of 3% NaCl water solution and kerosene (9:1 v/v), saturated with hydrogen sulphide; the volume of the mixture was 1 L and concentrations of the inhibitors used were in the rage of 50–150 mg ×L^-1^. Further, the rectangular (3.5 cm ×2 cm ×0.2 cm) steel samples were grazed with emery paper, washed with bidistilled water, degreased with acetone and ethanol, and dried at room temperature. After tests, the steel samples were washed again with bidistilled water, degreased with acetone and ethanol, dried and then weighed.

The corrosion rate (Kw (g ×m^-2^×h^-1^)), inhibition effectiveness (ηw , %), and surface coverage (θ) of the steel were determined both in the absence and presence of inhibitors 4a-d at various concentrations, using Equations (1), (2), and (3) [19,28]:

(1)KW=W1-W2Sxt

(2)ηW=[KWcorr.-KWcorr.(inh.)KWcorr.]x100%

(3)θ=KWcorr.-KWcorr.(inh.)KWcorr.

where *K_Wcorr_* and *K_Wcorr(inh.)_* are the corrosion rates in the absence and presence of inhibitor respectively, and W_1_ and W_2_ are weight losses of carbon steel in the absence and presence of the inhibitors, respectively.

### 2.3. Electrochemical measurements

Electrochemical corrosion studies were carried out on the Autolab PGSTAT 30 (Eco-Chemie, Netherlands) potentiostat, with 0.7 cm^2^ silver chloride electrode and platinum electrodes. The data obtained was processed using the GPES software. The above-described NaCl-water/kerosene-hydrogen sulphide mixture was used as a model solution at 25 °C, without stirring [11], while the concentration of the tested compounds varied from 50 to 150 mg ×L^-1^. The inhibition effectiveness (ηp, %) and surface coverage (θ) of steel corrosion were calculated by Equations (4) and (5):

(4)ηp=[icorr.-icorr.(inh)icorr.]x100%

(5)θ=icorr.-icorr.(inh.)icorr.

where i_corr.(inh.)_ and i_corr._ are the corrosion current density values in the presence and absence of inhibitor, respectively.

### 2.4. Scanning electron microscopy (SEM)

The surface morphology of the steel samples before and after the immersion was studied using the SEM–XL-30 microscope at the concentration of the inhibitors 4a-d 100 mg ×L^-1^ for 5 h in a mixture of 3% NaCl water solution and kerosene (9:1 v/v) saturated with hydrogen sulphide.

### 2.5. Biological activity tests

Antimicrobial activity of the synthesized compounds against SRB was studied at various concentrations (50, 75, 100, 150 and 200 mg ×L^-1^) of 4a-d using the method described in the literature [53]. Accordingly, compounds 4a-d were examined in water media containing SRB microorganisms (10^4^–10^6^ cells in 1 mL), which were taken from the oilfield “Chirag” (Baku, Azerbaijan). 3 mL from the prepared samples of SRB water solutions and studied compounds 4a-d were added to Postgate medium and were incubated for 15 days at 32 °C. After the incubation period, the concentration of hydrogen sulphide was determined. The control tests were carried out similarly, but without 4a-d. The degree of inhibition of SRB growth (S, %) was determined by Equation (6):

(6)S,%=C1-C2C1x100%

where C_1_ and C_2_ are concentrations (mg ×L^-1^) of hydrogen sulphide in control and inspected samples before and after incubation period, respectively.

## 3. Results and discussion

### 3.1. Characterization of the synthesized compounds

Quaternary piperidinium halides 4a-d were obtained by the reaction of 1-(4-(2-allylphenoxy) butyl) piperidine (3) with halides (HCl, HBr, HI, and ethyl iodide) (Scheme). It was revealed that the yields (66%–78%) and melting points (120 °C–145 °C) of the obtained compounds 4a-d vary, depending on the nature of the halide taken.

In our previous studies, it was shown that the length of the spacer (the length of the –CH_2_ group in the structure of dibromoalkane) affects the yield of the product [44,52]. Therefore, in our future studies, the syntheses were carried out using 1,4-dibromobutane. The formation of precursor compound 2 – 1-allyl-2-(4-bromobutoxy)benzene and 3 – 1-(4-(2-allylphenoxy) butyl) piperidine, and compounds 4a-d were confirmed by FTIR and NMR spectroscopy.

**Figure 1:**
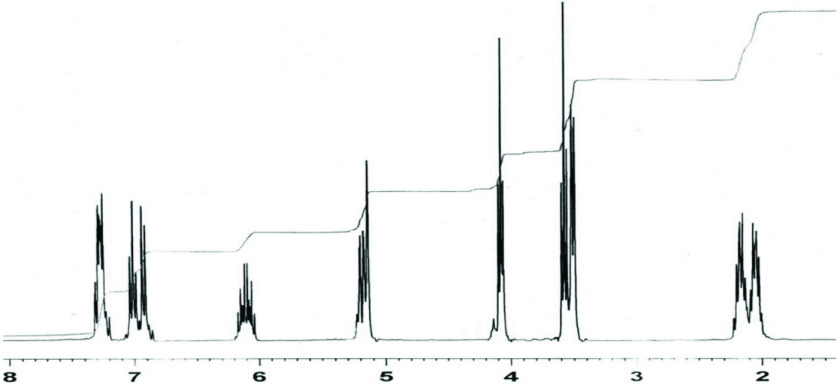
1H NMR spectra of 2.

^1^H NMR-spectra of 2 (CDCl_3_; δ , ppm) (Figure 1): 1.95–2.2 m (Br-CH_2_-C H_2_-C H_2_-CH_2_-O); 3.5 d (Ar-C H_2_-CH=CH_2_); 3.57 t (Br-CH_2-_); 4.1 t (–CH_2_-O-); 5.16 m (CH_2_=); 6.1 m (–CH_2_-C H =CH_2_); 6.95 m (C-H-arom); 7.25 d (C-H-arom).

**Figure 2:**
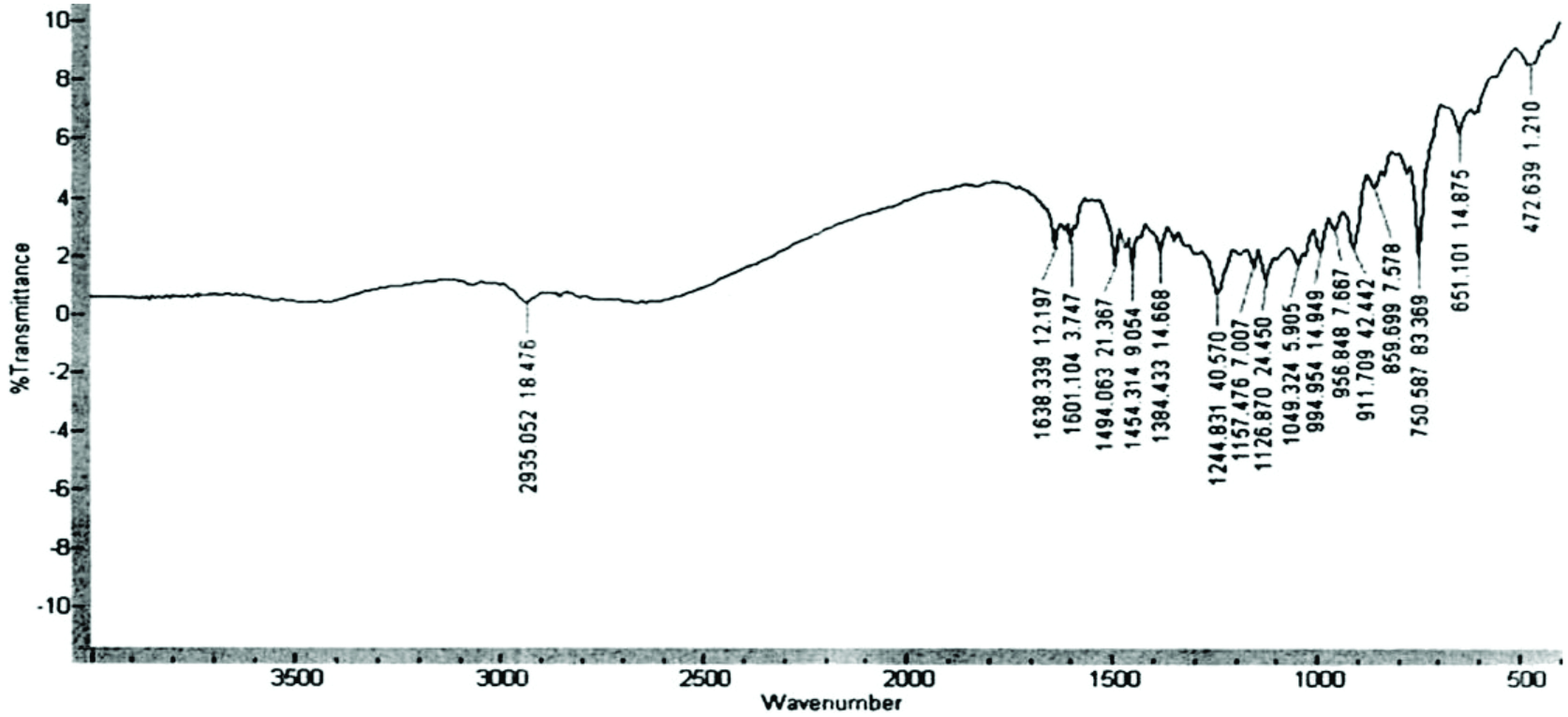
FTIR spectra of 3.

FTIR of compound 3 (Figure 2) showed the following absorption bands at 750 cm^-1^ (1,2 – substituted aromatic ring); 1244 cm^-1^ (C-O-deformation), 1494, 1601 cm^-1^ (C=C-arom), 3070 cm^-1^ (C-H-arom.); 1638 cm^-1^ (CH_2_=CH); 2935, 2970 cm^-1^ (CH_2_), and 1126 (C-N-stretching), as expected.

**Figure 3:**
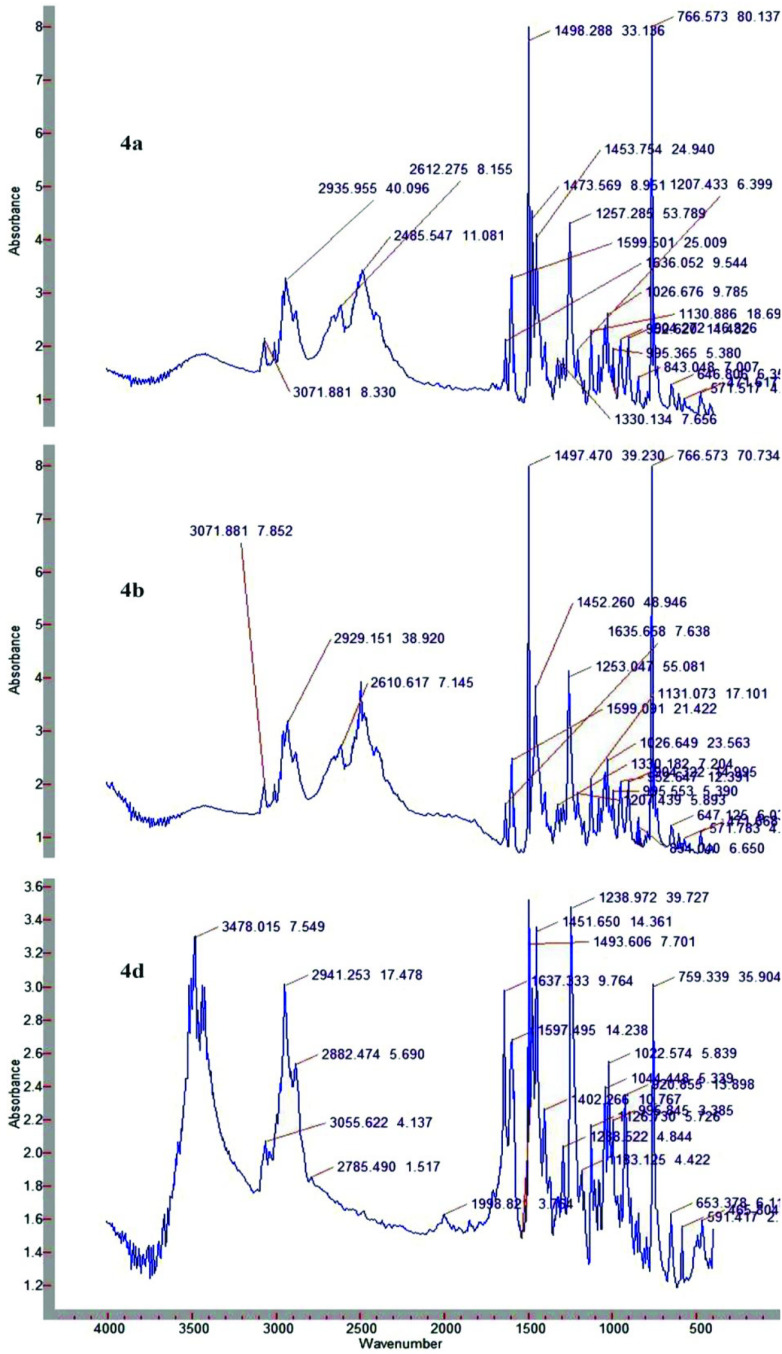
FTIR spectra of 4a, b, d.

FTIR of 4a (Figure 3) showed the different absorption bands at 766 cm^-1^ (1,2-substituted aromatic ring); 1257, 1026 cm^-1^ (Ar-O-C); 1498, 1473, 1451, 2935 cm^-1^ (CH_2_) ; 3071 cm^-1^ (C-H-arom); 1599 cm^-1^ (C=C-arom); 1636 cm^-1^ (CH_2_ = CH-); 1130 cm^-1^ (C-N stretching); 2485 cm^-1^ (N+H).

FTIR of 4b (Figure 3) showed the following absorption bands at 766 cm^-1^ (1,2-substituted aromatic ring); 1253, 1026 cm^-1^ (Ar-O-C); 2929, 1497, 1452 cm^-1^ (CH_2_) ; 3071 cm^-1^ (C-H-arom); 1599 cm^-1^ (C=Carom); 1635 cm^-1^ (CH_2_ = CH-); 1131 cm^-1^ (C-N- stretching), 2500, 2610 cm^-1^ (N+H).

FTIR of 4d (Figure 3) showed that at 759 cm^-1^ (1,2-substituted aromatic ring); 1238, 1022 (Ar-O-C); 2941, 1493, 1451 (CH_2_, CH_3_); 3055 cm^-1^ (C-H-arom); 1597 cm^-1^ (C=C-arom); 3478 cm^-1^ (C-H-stretching); 1238 (C-N- stretching).

**Figure 4:**
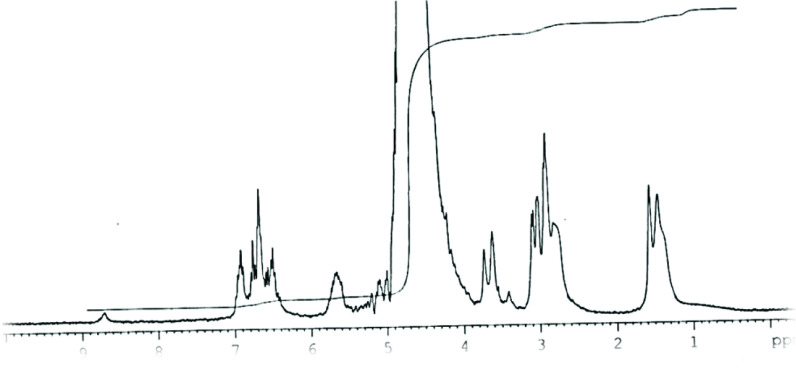
^1^H NMR spectra of 4b.

^1^H NMR of 4b (D_2_O; δ, ppm) (Figure 4): 1.5 m (2H, CH_2_) ; 2.85 d (2H, CH_2_ Ar); 3.05 t (2H, CH_2_N); 3.75 t (2H, CH_2_ O); 5.1 m (2H, =CH_2_) ; 5.7 m (1H, =CH); 6.4–6.9 m (4H, C_6_H_4_), 8.7 s (1H, +NH).

The results of the spectral characteristics confirmed the structure of the precursor compounds (2,3) and the synthesized (4a,b,d) compounds. In Figure 3, all the prepared compounds have the same absorption bands. The only difference is in the absorption band of 4d, owing to the presence of methyl group bands and ^+^NR _4_ in the structure. While comparing the ^1^H NMR spectra of compound 2 and 4d (Figure 1 and 4), it can be seen that the CH_2_ -Br signals have disappeared and replaced by the signals of the ammonium fragment, and also ^+^N-H in a weak field (8.7) appeared. While comparing the FTIR spectra of compound 3 (Figure 2) with compounds 4a,b (Figure 3), it can be seen that the absorption band of the quaternary ammonium salt appeared in spectra of 4a,b.

### 3.2. Gravimetric measurements

Organic compounds containing nitrogen, sulphur, oxygen, aromatic rings, as well as various multiple bonds in their structures are effective corrosion inhibitors, especially in acidic environments [54,55]. These compounds, due to the functional groups in the structures, are well adsorbed on the metal surface. Among these compounds are functionally substituted organic compounds based on alkenylphenol, which have higher corrosion inhibition properties in aggressive environments [28,29,44]. Moreover, in the presence of quaternary ammonium fragments in their structures, their inhibitory properties increase due to their good solubility in water [28].

Considering all these points, it was interesting to study of the synthesized 4a-d as corrosion inhibitors of steel St.3 in a mixture of 3% NaCl water solution and kerosene (9:1 v/v) saturated with hydrogen sulphide.

The obtained results (Table 1) demonstrated that the studied organic compounds 4a-d have inhibition effectiveness at various temperatures (25 °C, 35 °C, and 45 °C) and concentrations (50, 75, 100, 125, 150 mg ×L^-1^). Table 1 also shows that among the inhibitors compound, 4a has a relatively low protection degree. This can be explained based on the structure of the compound containing the chloride anion. Compounds 4b and 4d (150 mg ×L^-1^), containing bromide and iodide ions in the structures, had the best corrosion inhibition effectiveness as expected. Moreover, as it can be seen, the protection of steel was 93%–95% at 25 °C, 70%–72% at 35 °C, and 66%–68% at 45 °C. Compound 4c had a small difference in corrosion inhibition efficiency degree of protection (92% at the concentration 150 mg ×L^-1^) compared with 4b and 4d.

**Table 1:** Corrosion rate (K), surface coverage (θ), and corrosion inhibition effectiveness (η_w_, %) determined by gravimetric measurements at various temperatures for carbon steel.

Inhibitor	Conc., mg x L^-1^	K_T_ = 25◦C, g(m^2^x h)^-1^	K_T_=35◦C, g(m^2^x h)^-1^	K_T_=45◦C, g(m^2^x h)^-1^	θ_T_=25◦C	θ_T_=35◦C	θ_T_=45◦C	η_T_=25◦C,%	η_T_=35◦C, %	η_T_=45◦C, %
4a	50 75 100 125 150	2.394 1.862 1.374 0.975 0.709	4.062 - 3.148 - 2.234	4.357 - 3.560 - 2.657	0.46 0.58 0.69 0.78 0.84	0.20 - 0.38 - 0.56	0.18 - 0.33 - 0.50	46 58 69 78 84	20 - 38 - 56	18 - 33 - 50
4b	50 75 100 125 150	1.723 1.374 0.842 0.532 0.310	3.402 - 2.184 - 1.523	3.985 - 2.604 - 1.807	0.61 0.69 0.81 0.88 0.93	0.33 - 0.57 - 0.70	0.25 - 0.51 - 0.66	61 69 81 88 93	33 - 57 - 70	25 - 51 - 66
4c	50 75 100 125 150	1.596 1.197 0.754 0.576 0.355	3.351 - 2.285 - 1.625	3.826 - 2.657 - 1.860	0.64 0.73 0.83 0.87 0.92	0.34 - 0.55 - 0.68	0.28 - 0.50 - 0.65	64 73 83 87 92	34 - 55 - 68	28 - 50 - 65
4d	50 75 100 125 150	1.285 0.797 0.576 0.399 0.222	3.148 - 1.930 - 1.422	3.879 - 2.391 - 1.700	0.71 0.82 0.87 0.91 0.95	0.38 - 0.62 - 0.72	0.27 - 0.55 - 0.68	71 82 87 91 95	38 - 62 - 72	27 - 55 - 68
Without inhibitor	-	4.33	5.07	5.314	-	-	-	-	-	-

Furthermore, the results of the studies show that compounds 4b and 4d at the concentration 150 mg ×L^-1^ have high inhibition properties and that the studied inhibitors, due to their properties, are not inferior to corrosion inhibitors, which are known in the literature [8], and in some cases even surpassed them [1].

### 3.3. Potentiodynamic polarization studies

**Figure 5:**
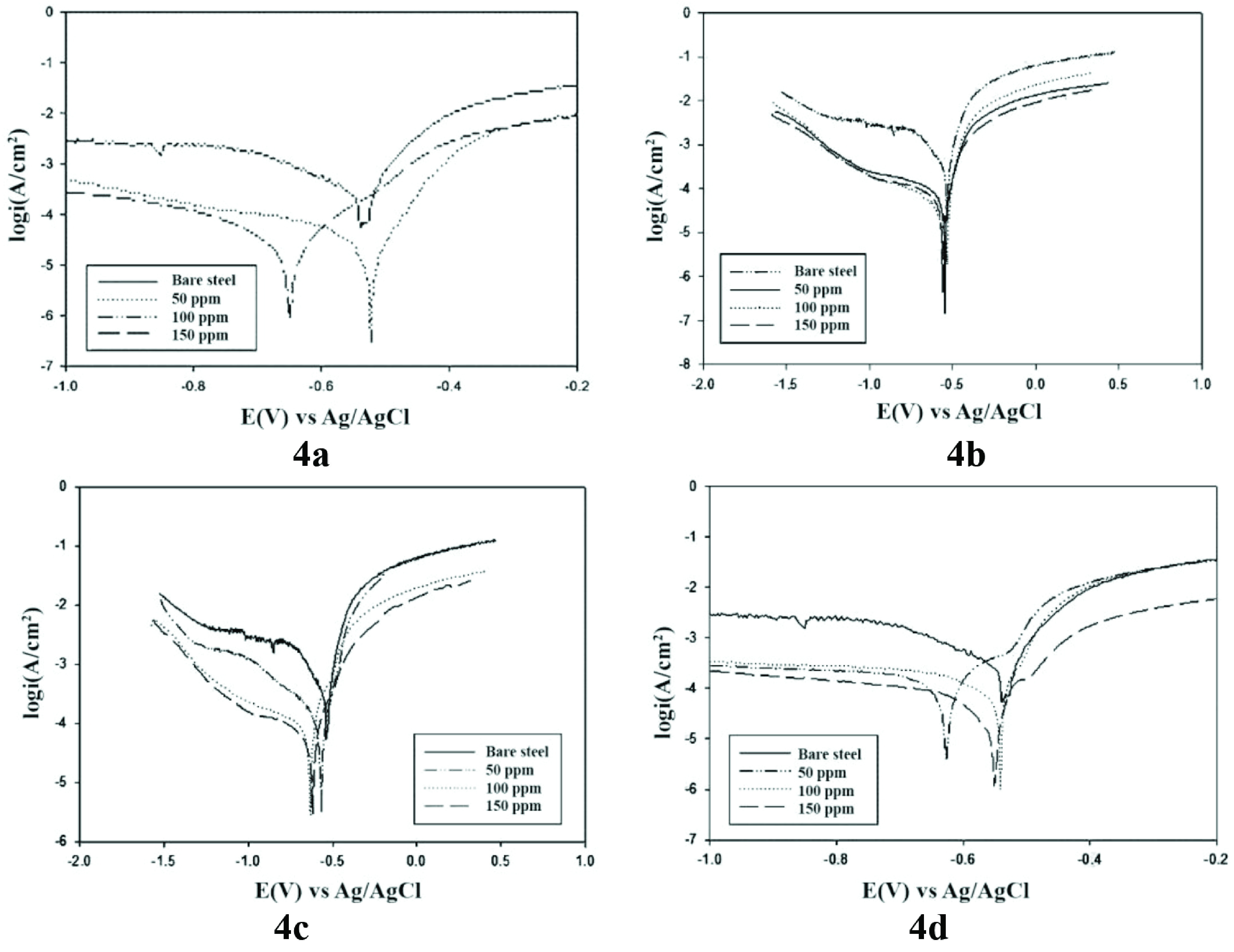
Polarization curves of carbon steel in the model system saturated with H_2_S and containing 4a-d at the different concentrations.

The potentiodynamic polarization studies (Figure 5) revealed a shift in currents. With an increase in concentration of 4a-d, the corrosion potential shifts towards positive values. This can be the result of the adsorption of the tested compounds on the surface of the steel electrode. In this case, the rate of redox reactions slows down, and inhibition of corrosion process occurs. Notably, the comparison of the results of electrochemical and gravimetric studies conducted at 25 °C makes it possible to identify the correlation.

The obtained values of the corrosion current densities (i_corr._), Tafel slopes (β_c_,βsub>a), corrosion rate (K), surface coverage (θ) , and percentage of inhibition effectiveness (η_p_, %) of the synthesized compounds are given in Table 2. These data demonstrate that the studied inhibitors shift the corrosion potential (E_corr._) into the cathodic direction. The cathodic Tafel slopes (β_c_) and anodic Tafel slopes (β_a_) values depend on the concentration of 4a-d. Among the investigated substances, high corrosion effectiveness was exhibited by substances 4b, 4c, and 4d at concentrations 100–150 mg ×L^-1^ with inhibition effectiveness as 68%–92%.

**Table 2:** 

Inhibitor	Conc. of inh., mg x L^-1^	E, V	I, μ Acm^-2^	β_c_	β_a_	R_p_Ω x sm^-2^	θ	η_p_%
4a	50 100 150	--0.62 --0.54 -	78 71 -	0.13 0.37 -	0.06 0.03 -	234 160 -	0.40 0.45 -	40 45 -
4b	50 100 150	--0.54 --0.55 --0.57	42 24 10	0.17 0.17 0.03	0.07 0.10 0.04	513 611 701	0.68 0.82 0.92	68 82 92
4c	50 100 150	--0.57 --0.63 --0.62	34 25 20	0.04 0.07 0.06	0.07 0.04 0.04	357 412 557	0.74 0.81 0.85	74 81 85
4d	50 100 150	--0.62 --0.55 --0.56	25 16 11	0.12 0.11 0.07	0.09 0.06 0.06	887 1066 1191	0.81 0.88 0.92	81 88 92
Without inhibitor	-	--0.54	130	0.03	0,05	64	-	-

The results of anticorrosion studies showed that the studied inhibitors are not inferior to the inhibitors described in the literature in terms of anticorrosion properties [3–5].

### 3.4. Adsorption isotherm and thermodynamic data

**Figure 6:**
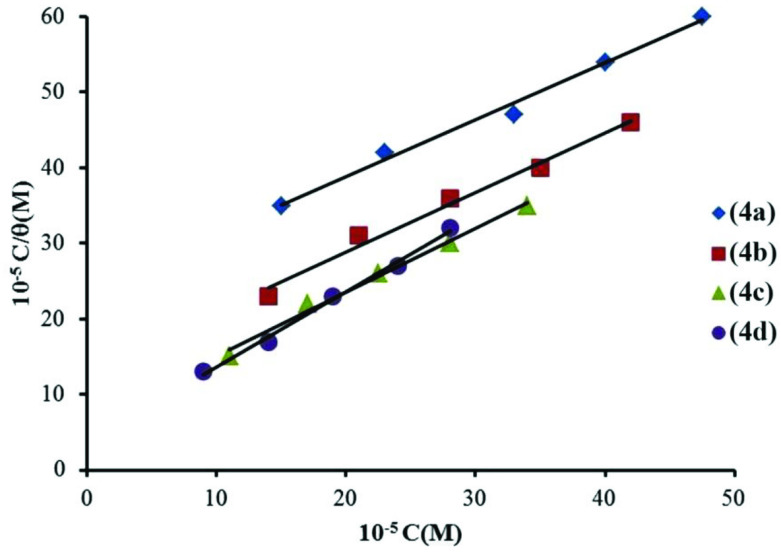
Langmuir adsorption plots for carbon steel at different concentrations of 4a-d at 25 °C.

The analysis of adsorption isotherms can provide basic information on the interaction of an inhibitor with a metal surface [11]. Therefore, the adsorption ability on the metal surface was studied. The surface coverage values (θ) were determined at different concentrations of 4a-d from gravimetric measurements (Figure 6).

The plots C/θ versus C (Figure 6) are linear with correlation coefficients 0.9972, 0.9919, 0.9983, and 0.9991, respectively. In this case, the adsorption of indicators on the carbon steel surface obeys the Langmuir adsorption isotherm by Equation (7):

(7)Cθ=C+1Kads

where C is the inhibitor concentration, θ is the degree of coverage on the metal surface and K_ads_ is the equilibrium constant for adsorption desorption process, calculated by the reciprocal of the intercept of isotherm line at various temperatures (Table 3).

**Table 3:** Thermodynamic parameters of the inhibitor 4a-d adsorption on the surface of carbon steel in the presence of hydrogen sulphide, at different temperatures and concentrations.

Inhibitor	Temperature (° C)	K_ads_ (x 10^3^ M^-1^	Δ G_ads_ kJ x mol^-1^	Δ H_ads_ kJ x mol^-1^	Δ S_ads_ J mol^-1^ x K^-1^
4a	25 35 45	4.11 1.29 1.15	-30.57 -28.62 -29.25	-50.53	66.99 71.12 66.90
4b	25 35 45	7.65 3.03 1.98	-32.11 -30.82 -30.68	-53.46	71.66 73.52 71.62
4c	25 35 45	13.40 4.56 3.31	-33.49 -31.86 -32.05	-55.36	73.37 76.30 73.31
4d	25 35 45	21.47 6.18 3.48	-34.66 -32.64 -32.18	-71.87	124.89 127.39 124.83

It was found that the values of the equilibrium constant decrease with increasing temperature. The high values of K_ads_ (4.11· 10^3^M^-1^, 7.65· 10^3^M^-1^, 13.40· 10^3^M^-1^, and 21.47· 10^3^M^-1^ for compounds 3a, 3b, 3c, and 3d, respectively at 25 °C) reflect the high adsorption ability of the inhibitors on the carbon steel surface.

The free energy (Δ *G_ads_* ) of adsorption can be calculated by Equation 8:

(8)ΔGads.=-KTln(55.5Kads)

where *ΔG_ads_* is the free energy of adsorption, *K_ads_* is the equilibrium constant, *T* is the absolute temperature, and 55.5 is the molar concentration of water.

The negative values of *ΔG_ads._* (–30.57, –32.11, –33.49, and –34.66 kJ ×mol^-1^ for 3a, 3b, 3c, and 3d, respectively) indicate spontaneous adsorption of the prepared surfactants on the carbon steel surface.

The *ΔH_ads_* values (–50.53, –53.46, –55.36, –71.87 kJ ×mol^-1^ for inhibitors 3a, 3b, 3c, and 3d, respectively) were calculated using Kads. at respective temperatures. The *ΔH_ads_* values indicate that the adsorption in hydrogen sulphide area is an exothermic process. Entropy of the inhibitors’ adsorption ( *ΔS_ads_* ) was calculated by Equation (9):

(9)ΔGads=ΔΔHads.-TΔSads.

The positive values of *ΔS_ads_* (66.99, 71.66, 73.37, 124.89 J ×mol^-1^×K^-1^ for inhibitors 3a, 3b, 3c, and 3d, respectively) are possibly related to an increase in disorder due to the adsorption of the studied molecules and desorption of water molecules.

### 3.5. Scanning electron microscopy (SEM)

**Figure 7:**
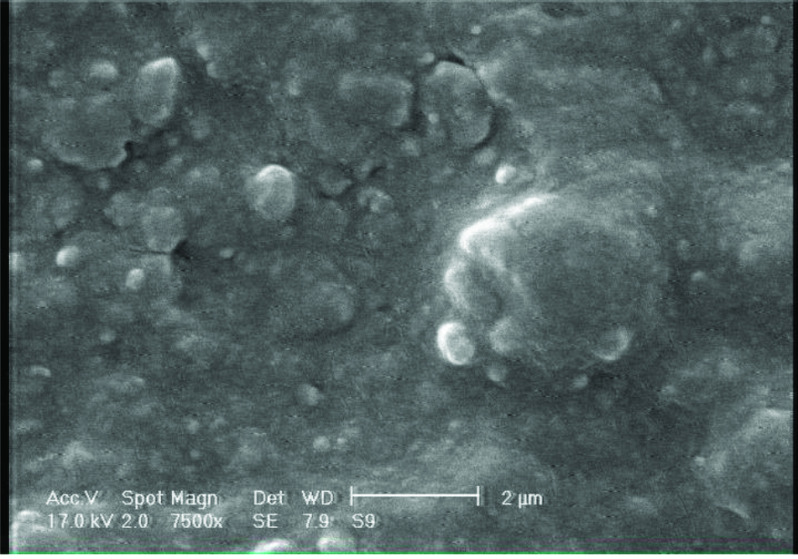
SEM image of surface of carbon steel after immersion for 5 h, 25 °C.

**Figure 8:**
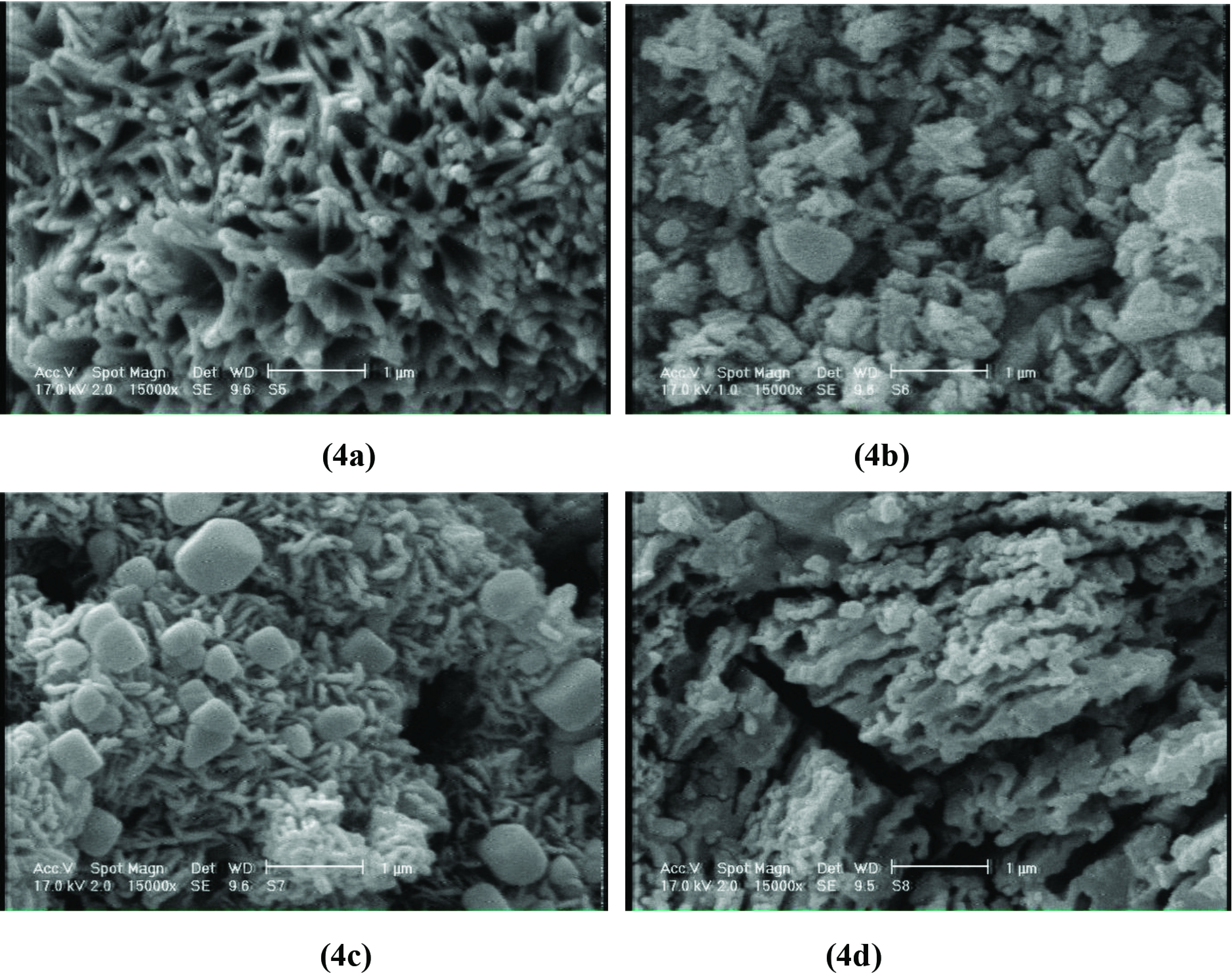
SEM images of surface of carbon steel in the presence of 100 mg ×L^-1^ inhibitors 4a-d for 5 h, 25 °C.

To test the inhibitory properties of organic compounds, the morphology of the metal surface is important [8]. For this purpose, the surface morphology of steel in the absence and presence of inhibitors 4a-d after immersion for 5 h was studied. The results are shown in Figures 7 and 8.

It was thus revealed that the surface was strongly damaged (Figure 7) in absence of the inhibitors 4a-d. Figure 8 demonstrates that the investigated compounds were adsorbed on the steel surface and protect the surface in acidic solution at low concentration (100 mg ×L^-1^), confirming their high inhibition effectiveness.

### 3.6. Antimicrobial activity

To prevent biocorrosion of equipment that is used in the oil industry, various compounds containing active atoms and functional groups in their structures are used [7–10]. Considering this, compounds 4a-d were studied as biocides for SRB microorganisms at the concentration 50–200 mg ×L^-1^. The results are given in Table 4. According to the experimental results of the antimicrobial activity of 4a-d against SRB, all the compounds showed bactericidal activity. As shown earlier, the bactericidal activities of the studied compounds depend on their chemical structures. For example, compound 4a, consisting in the structure chloride ions, has 89% antimicrobial activity at the concentration 200 mg ×L^-1^. However, 4b with bromide anions in the structure has 100% degree at 150 mg ×L^-1^. The most actives are 4c and 4d. These compounds have 100% degree at 100 mg ×L^-1^, probably due to the content of iodide ions in the structures of 4c and 4d. In general, the studied compounds have better bactericide effectiveness in comparison with those known [7,10,56].

**Table 4:** Antimicrobial activity of 4a-d against SRB.

Inhibitors	The degree (%) of inhibition of growth of SRB at the concentration of inhibitors, mg ×L^-1^				
50	75	100	150	200
4a	45	62	71	79	89
4b	81	90	96	100	100
4c	88	94	100	100	100
4d	91	95	100	100	100

## 4. Conclusions

1-(4-(2-Allylphenoxy)butyl)piperidin-1-ium halides 4a-d were synthesized by the reaction of 1-(4-(2-allylphenoxy) butyl)piperidine with hydrogen halides and ethyl iodide. The compounds were characterized by NMR and FTIR spectra. Synthesized compounds 4a-d (50–150 mg ×L^-1^) were studied as corrosion inhibitors in acidic media using the gravimetric and potentiometric methods. It was demonstrated that 4a-d have high anticorrosion effectiveness for steel St.3 at concentration 150 mg ×L^-1^ by gravimetric (84%–95%) and potentiometric (85%–92%) method. The evaluated thermodynamic parameters of adsorption show that compounds 4a-d are strongly adsorbed on the carbon steel surface, while the SEM images demonstrate good coverage of the surface. Therefore, the high corrosion inhibition effectiveness can be related to the adherent adsorption of these cationic surfactants on the steel surface and formation of a protective film.

Moreover, 4a-d were studied as reagents against SRB and their high bactericidal activity (100%) was revealed at concentration 100 mg ×L^-1^.

Thus, our study allowed us to conclude that compounds 4a-d can be used as corrosion inhibitors and biocides. The results show that using such compounds can reduce the rate of metal corrosion and increase their service life.
